# Radiofrequency therapy improves exercise capacity of mice with emphysema

**DOI:** 10.1038/s41598-021-99474-8

**Published:** 2021-10-08

**Authors:** Mai Tsutsui, Chung Yan Cheung, Takeyuki Wada, Jen-erh Jaw, Cheng Wei Tony Yang, Pascal Bernatchez, Zoe White, Chen Xi Yang, Eun Jeong Annie Bae, Lauren H. Choi, Dan Gelbart, Samuel Lichtenstein, Lindsay Machan, Eran Elizur, Kim Wolff, Evan Goodacre, Marek Lipnicki, Denny Wong, Don D. Sin

**Affiliations:** 1 Centre for Heart Lung Innovation, St. Paul’s Hospital, University of British Columbia, 1081 Burrard Street, Vancouver, BC V6Z 1Y6 Canada; 2 Ikomed Technologies Inc, Vancouver, BC Canada; 3 Division of Respiratory Medicine, University of British Columbia, Vancouver, BC Canada; 4 Department of Anesthesiology, Pharmacology and Therapeutics, University of British Columbia, Vancouver, BC Canada; 5 Division of Cardiac Surgery, University of British Columbia, Vancouver, BC Canada

**Keywords:** Diseases, Medical research

## Abstract

Emphysema is a common phenotype of chronic obstructive pulmonary disease (COPD). Although resection of emphysematous tissue can improve lung mechanics, it is invasive and fraught with adverse effects. Meanwhile, radiofrequency (RF) treatment is an extracorporeal method that leads to tissue destruction and remodeling, resulting in “volume reduction” and overall improvement in lung compliance of emphysematous lungs. Whether these changes lead to improved exercise tolerance is unknown. Here, we investigated the effectiveness of RF treatment to improve the exercise capacity of mice with emphysema. Fifty-two mice (7 weeks of age) were used in this experiment. A bilateral emphysema model was created by intratracheally instilling porcine pancreatic elastase (PPE) (1.5U/100 g body weight). RF treatment (0.5 W/ g body weight) was administered extracorporeally 14 days later and mice were sacrificed after another 21 days. The exercise capacity of mice was measured using a treadmill. Treadmill runs were performed just before PPE instillation (baseline), before RF treatment and before sacrifice. Following sacrifice, lung compliance and mean linear intercept (Lm) were measured and fibrosis was assessed using a modified Ashcroft score. There were 3 experimental groups: controls (instilled with saline, n = 12), emphysema (instilled with porcine pancreatic elastase, PPE, n = 11) and emphysema + treatment (instilled with PPE and given RF, n = 9). At endpoint, the maximum velocity of the emphysema + treatment group was significantly higher than that of the emphysema group, indicating improved exercise tolerance (86.29% of baseline vs 61.69% of baseline, p = 0.01). Histological analysis revealed a significant reduction in emphysema as denoted by Lm between the two groups (median 29.60 µm vs 35.68 µm, p = 0.03). The emphysema + treatment group also demonstrated a higher prevalence of lung fibrosis (≧Grade 3) compared with the emphysema group (11.7% vs 5.4%, p < 0.01). No severe adverse events from RF were observed. RF treatment improved the exercise capacity of mice with emphysema. These data highlight the therapeutic potential of RF treatment in improving the functional status of patients with COPD.

Chronic Obstructive Pulmonary Disease (COPD) is a common inflammatory disorder of the lung, characterized by persistent airflow limitation. In COPD, the airways become progressively narrowed, resulting in impaired lung function, increased work of breathing, and shortness of breath. Currently, COPD affects over 300 million people worldwide and is the leading cause of hospitalizations in many industrialized countries such as the United States (US) and Canada. COPD is responsible for over 4.5 million deaths annually and is one of the major causes of mortality worldwide^1,2^. Typically, smoking-related COPD is accompanied by emphysema, which is characterized by the destruction of lung tissue resulting in the loss of alveolar integrity, enlargement of the alveolar spaces, poor gas exchange, and airway collapse due to the loss of elastic recoil^3^. This remodeling results in impairment in airflow and hypoxemia.

Lung volume reduction surgery (LVRS) is a potential treatment for COPD, but it is limited to a subpopulation of patients with severe emphysema involving the upper lobes and with low exercise capacity. LVRS has been demonstrated to reduce breathlessness, improve exercise capacity and lung function, and prolong survival^4,5^. The physiologic basis for this treatment is that by selectively removing the most diseased segments of the lung, the lung deflates and there is better matching of ventilation and (blood) perfusion, leading to less gas trapping and improved gas exchange, respectively, during exercise^6^. However, LVRS is invasive and fraught with considerable perioperative morbidity and mortality related to the procedure. As such, very few sites currently perform LVRS^2,3,7^. To overcome the high invasiveness and the cost of LVRS, endoscopic lung volume reduction (ELVR) including bronchoscopic lung volume reduction (BLVR) has been developed using one-way valves, coil implants (LVR coil, LVR-C), sealants/hydrogels (BioLVR), airway bypass stents, and bronchial thermal vapor ablation (BTVA) therapy. However, these methods are still invasive and expensive and fraught with certain adverse effects including hemoptysis and pneumothorax^8–12^. According to a multicenter randomized controlled trial to evaluate endobronchial valves (EBV), 26.6% (34/128) of EBV subjects developed pneumothorax and four deaths occurred (4/128)^8^. Moreover, it is still difficult to determine the patients who benefit from ELVR^9,10^.Thus, minimally-invasive (or ideally completely non-invasive) modalities to deliver the benefits of LVRS are still clinically needed.

Radiofrequency (RF) is commonly used to treat solid tumors and atrial fibrillation in a procedure called RF ablation (RFA)^12,13^. The principle of RFA is that RF waves agitate water molecules in the surrounding tissue, producing friction and heat, which in turn induce cellular death via coagulation and necrosis^12^. Healthy regions with good blood supply are relatively spared from thermal damage because the circulating blood can absorb the external heat and carry it away via a process known as a "heat sink effect”^14^. A well-known pathologic feature of emphysema is pulmonary vascular remodeling, which causes diminution of blood flow to the emphysematous regions^15^, making these areas susceptible to thermal injury. Based on these concepts, we applied RF therapy extracorporeally to destroy emphysematous regions of the COPD lung in a rodent model of emphysema. Previously, we showed that the application, using external electrodes, of RF energy in the form of electromagnetic waves improved lung compliance by selectively heating pulmonary emphysematous tissue and inducing mild fibrosis in the affected lung while sparing normal lung regions^16^. However, in that study, we did not determine whether the physiological changes led to improvements in the functional status of animals with emphysema which would a critical indicator of a beneficial therapeutic effect. Here, we investigated the effectiveness of extracorporeal RF treatment in improving the exercise capacity of mice with emphysema.

## Methods

The study was conducted in compliance with the ARRIVE guidelines (https://arriveguidelines.org/).

### Animals

Seven-week-old male C57BL/6 mice (n = 52), weighing around 22 g, from Jackson Laboratories (ME, USA) were used. All animals were housed in a temperature- and humidity-controlled (temperature at 22 ℃ ± 4 ℃ humidity 55% + /- 15%) environment under a 12-h light/dark cycle with food and water available ad libitum at the St. Paul’s Hospital animal facility in Vancouver BC. Animals were housed as a group in static cages. For bedding, each cage contained a mix of Bed-O-Cob, Enviro-Dry paper-strands nesting material, a nestled, and an igloo/house for nesting and shelter. Cages and reverse osmosis water bottles were changed once per week or more often as needed. Food, water levels, and animals were observed at a minimum once daily. This study was approved by the Animal Care Committee of the University of British Columbia (A19-0307). The care and handling of the animals were in accordance with the policies of the Canadian Council on Animal Care.

### Experimental groups

**Figure 1:**
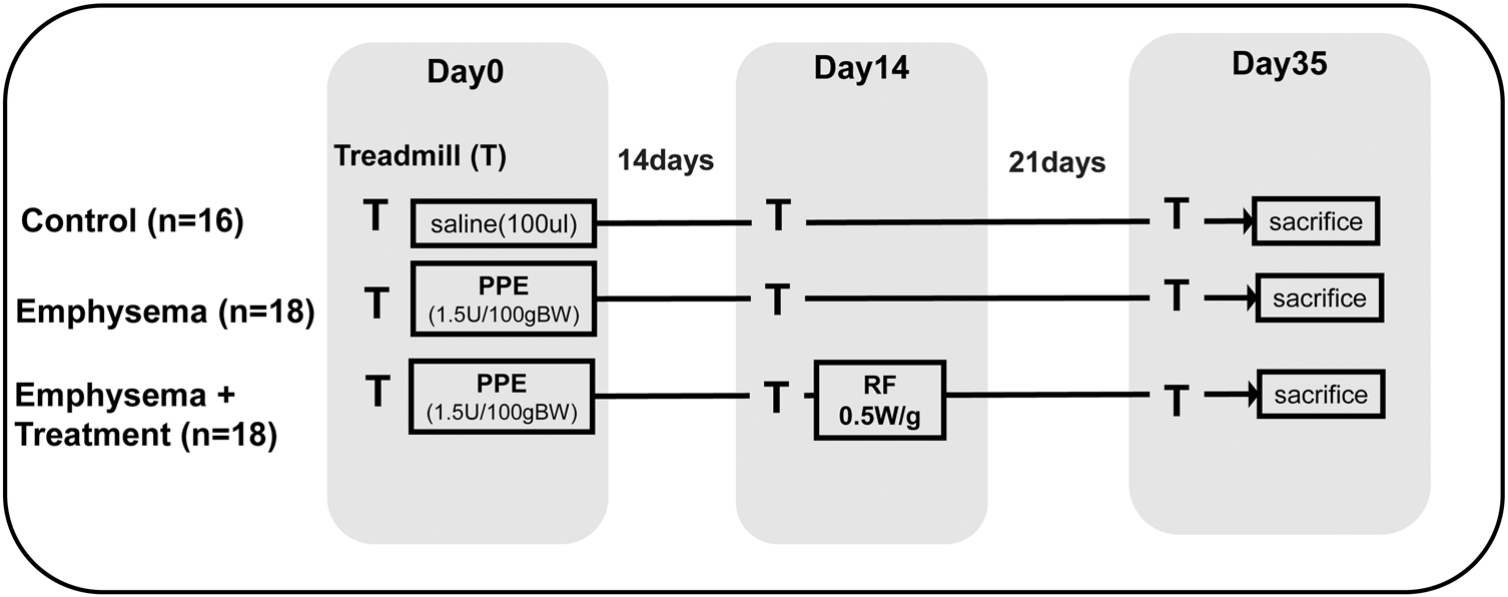
Timeline of the experimental protocol. Fifty-two mice (7 weeks of age) were used in this experiment. We first induced bilateral emphysema in the lungs by instilling porcine pancreatic elastase (PPE) (1.5U/100 g body weight (BW)) into the lungs of mice. Radiofrequency (RF) (0.5 W/ g BW) treatment was then performed two weeks later and mice were sacrificed three weeks post-RF treatment. The exercise capacity of mice was determined on a treadmill test. Treadmill runs were performed just prior to PPE instillation (baseline or day 0), before RF treatment (day 14) and on the day of sacrifice (day 35). Following sacrifice, lung compliance, mean linear intercept (Lm) and fibrosis were assessed.

The animals were randomly divided into three groups: (A) controls, which were exposed to intratracheal saline (100 μl; n = 16); (B) emphysema group, which was exposed to intratracheal porcine pancreatic elastase (PPE) (100 μl; n = 18); and (C) emphysema + treatment group, which was exposed to intratracheal PPE (100 μl) + RF treatment (n = 18) (Fig. 1).

### Treadmill exercise test

A digitized DigiGait treadmill (Mouse Specifics, Inc. MA, USA) was used to measure the exercise capacity of mice. Mice were placed in the chamber and after 1 min of acclimation, the treadmill was activated at a velocity of 13.1 cm/sec. Velocity was increased by 2 cm/sec every 2 min until mice could no longer keep up with the pace. In the event the mice did not reach a peak velocity of at least 13.1 cm/sec, treadmill speed was reduced to 5 cm/sec for additional acclimation, after which the protocol was reactivated. No stimulatory agents (e.g. shock or candy) were used to motivate the mice. Peak velocity was determined as the maximal speed reached by the mice during each treadmill test. Maximum velocity for each run was compared to the baseline value for each mouse.

The treadmill test was performed in the morning at day 0 (i.e. the day of intratracheal instillation), day 14 (i.e. 2 weeks after the intratracheal instillation), and day 35 (i.e. 5 weeks after the intratracheal instillation of PPE and 3 weeks post-RF therapy).

### Emphysema induction

For PPE instillation, mice were first anesthetized with 2–4% isoflurane + 1.5 L/min 100% oxygen. This was supplemented with intraperitoneal injections of ketamine hydrochloride (25 mg/kg body weight; Narketan, VETOQUINOL N.-A. INC. QC, Canada) and xylazine hydrochloride (3.5 mg/kg body weight; X1251-1G, Sigma Aldrich, MS, USA). Following anesthesia, the mice were mounted on a work stand (Hallowell EMC, MA) at a 45-degree angle. Using a laryngoscope (LS-1; PennCentury, Philadelphia, PA), we introduced a microsprayer (type IA-1c; PennCentury, Philadelphia, PA, USA) into the oropharynx of these mice and delivered porcine pancreatic elastase (PPE; Sigma, Aldrich, Taufkirchen, Germany) into the lungs. Mice were given a solution of PPE (1.5U/100 g body weight), which was diluted in 100 uL of normal saline solution. The mice were then placed on a heating pad and given 1 ml of warm saline subcutaneously. The dose of PPE was determined based on dose optimization experiments shown in the supplement files. We found that a PPE dose of 1.5U/100 g body weight was sufficient to induce emphysema at 2 weeks post-instillation (see Supplementary Fig. 1: Figure S1a-d).

### Radiofrequency treatment

**Figure 2:**
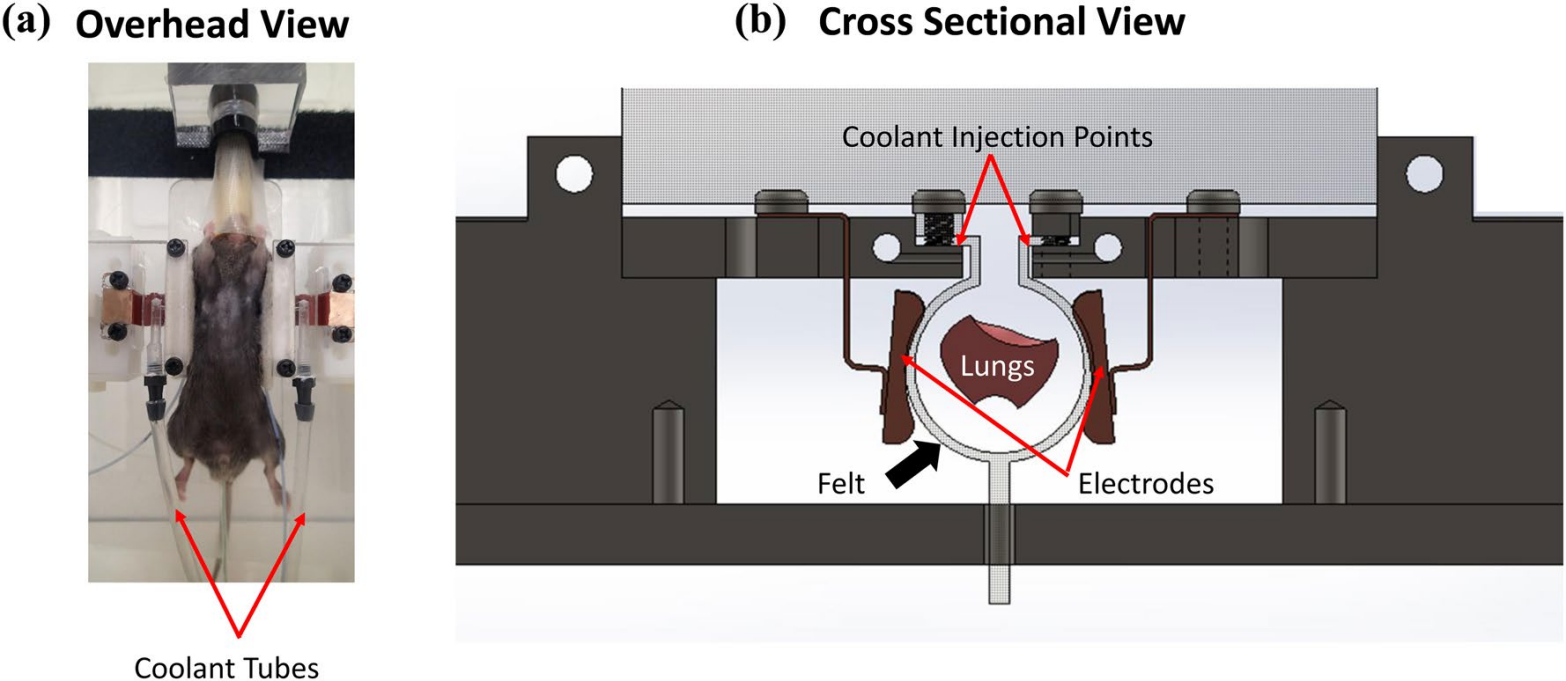
Gross image of the RF device. (**a**) The overhead view of the mouse positioning. The mouse was set between the electrodes and was supported by a felt over which cool saline was dispensed during the RF treatment. (**b**) The cross-sectional view of the RF device. The electrodes were designed to match the position of the lungs.

Two weeks after the PPE instillation (Day 14), mice were anesthetized with 2–4% isoflurane + 1.5L/min of 100% oxygen. After shaving off the dorsal fur of the mice, the animals were anesthetized with 0.5–4% isoflurane anesthesia and placed in a supine position. The mice’s internal temperature was monitored using a rectal thermometer throughout the experiment (ATK-610B Single Input K-Type Thermometer, ATP). The RF device (Fig. 2) had 2 independent electrodes (18 mm × 15 mm) to treat separately the left and right lungs. A felt pad was designed to support and protect the mice from direct heating by the RF electrodes. The external temperature of the mice was monitored using 2 thermometers, which were embedded into the felt pad. To avoid thermal injury to the skin related to RF therapy, cold distilled water (5 ℃-15 ℃) was infused over the covering felt pad at a rate of 50 ml/minute, while the RF device was activated. The RF device consisted of two parts: a 13.56 MHz RF Power Source capable of 100 W output, and a matching network. The amplifier was made by T&C Power Conversion of Rochester, N.Y and the model number is 0113. Two electrode probes, which conduct RF waves from the device, delivered RF to the chest wall of mice. Mice were settled into the RF device for impedance measurements. Low power RF (0.5 W/g body weight) was administered for 1-min and repeated 3 times at 30-s intervals. The power was determined after conducting pilot safety investigation tests (see Supplementary Fig. 2: Figure S2). RF was stopped whenever the surface temperature reached 42C degrees, and the next cycle of RF was initiated after a 30-s pause and after the surface temperature declined below this threshold. RF therapy could not be continued in two animals because their temperature exceeded this threshold during the first RF cycle. As these mice received “insufficient RF”, they were excluded from the final analysis. Post-RF therapy, the mice were monitored at the following time points: (1) right after RF treatment, (2) 6 h post-treatment, (3) daily for 5 days post-treatment.

### Compliance measurement- water displacement method

Lung compliance was measured using a water displacement method, following Archimedes’ principle, which has been described previously^17^. Briefly, the harvested lung was connected to a soft plastic tube through a tracheal catheter and completely submerged in water. Lung compliance was calculated as ΔVolume/ΔPressure^18^. The deflation limb from 15 cmH_2_O to 0 cmH_2_O was used to calculate lung compliance in this experiment.

### Histological analysis

Following euthanasia, the lung was instilled with 10% formalin through a tracheal catheter at 20 cmH_2_O pressure. The formalin-filled lung was submerged into 10% formalin for 2–5 days. Both left and right lungs were then embedded in 4% agar in order to preserve structure before cutting into trans-axial 4 mm slices for paraffin stabilization^19^. The specimen was stained with hematoxylin and eosin (H&E) and Masson's trichrome. Stained slides were scanned and analyzed using the Aperio (Aperio Technologies; Vista, CA) scanning system. The extent of emphysema was estimated by measuring the mean linear intercept length (Lm) using STEPanizer^20^. We first divided the HE stained lungs into multiple 1 mm^2^ regions by placing axis and grids in the Aperio. We numbered all 1 mm^2^ squares (i.e. total X squares). By inserting that number (X) in the program, we obtained a random number (Y) from Random.org (https://www.random.org). Starting from that randomly selected number (Y), we selected 10 images as (Y + X/10, Y + 2X/10 …). Integer numbers were used in this formula. Lm was calculated from the length of the lines shown in the STEPanizer multiplied by the number of the lines divided by the sum of all counted intercepts.

The Masson’s trichrome-stained specimens were used for fibrosis scoring. Each lung section was divided into 1 mm^2^ regions as per the Lm calculation, and the total lung was evaluated for fibrosis, which was performed using a modified Ashcroft Scale^21^. Each square region was given a grade from 0–8 upon evaluation at 8 × and 20 × magnification and the frequency distribution of grades was determined by calculating the proportion of fibrosis grade ≧ 3 (which was derived by dividing the number of squares that had scores of 3 or above by the total number of squares for the whole lung). The fibrosis scoring was performed by 2 different individuals who were blinded to the group assignment of the mice.

### Statistical analysis

All results were expressed as the mean value ± standard error of the mean (SEM) unless otherwise indicated. Mean value was adopted for results which passed the normality test and data which did not pass are shown as median. Where applicable, data were analyzed using either an unpaired T test or Mann–Whitney U test. The comparison of the frequency distribution was performed by a chi-square test. The primary endpoint of the study was the change in the peak velocity on a maximal treadmill test at 35 days post-intratracheal instillation of PPE from baseline between mice in the emphysema alone and emphysema + treatment groups (primary analysis). As there was considerable variation in the peak velocity across mice, the changes were expressed as percentage (%) of baseline value for each animal. All analyses were conducted using GraphPad Prism 5.0 (GraphPad Software, Inc., San Diego, CA) and a value of p < 0.05 was considered statistically significant.

### Ethics approval and consent to participate

This study was approved by the Animal Care Committee of the University of British Columbia (A19-0307). The care and handling of the animals were in accordance with the policies of the Canadian Council on Animal Care.

## Results

### Mortality

**Figure 3:**
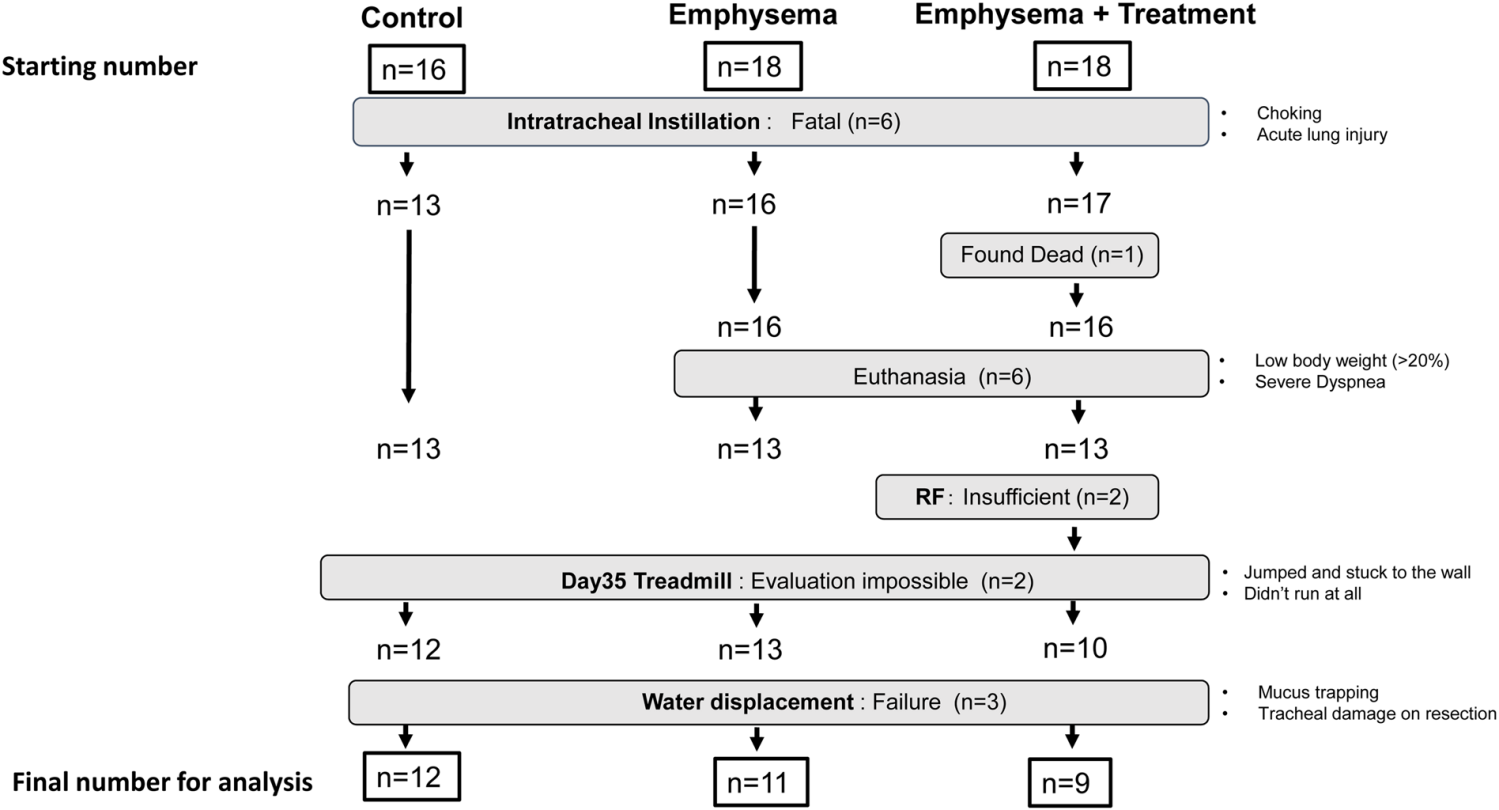
The flow of the study.

A flow diagram of the study is shown in Fig. 3. During the instillation process, 3 mice in the saline group, 2 mice in the emphysema group, and 1 mouse in the emphysema + treatment group died immediately following intratracheal instillation from asphyxiation or shock. One mouse died 7 days after the PPE instillation in the emphysema + treatment group. Six mice were euthanized before reaching their clinical endpoint for the following reasons: four (2 each in the emphysema and emphysema + treatment group) experienced over 20% body weight loss and 2 mice in the emphysema group became ill during the course of the experiment. Euthanasia was determined based on a clinical health scoring system approved by the Animal Care Committee of the University of British Columbia. No deaths were related to RF therapy.

We excluded 2 mice in the functional analysis owing to poor performance on the treadmill test (these mice did not run at all on the treadmill), 2 mice that received “insufficient RF”, and 3 mice owing to failures in the water displacement method (related to mucus trapping and tracheal damage). The final sample size in each group was (A) control (n = 12); (B) emphysema (n = 11); and (C) emphysema + treatment (n = 9).

### Body weight

Mice in both emphysema and emphysema + treatment groups, showed greater body weight loss compared to the saline control group until day 7 post-instillation. Weight loss was the most severe on day 2 (control vs emphysema vs emphysema + treatment; 99.61 ± 1.57% baseline vs 88.66 ± 1.96% baseline vs 89.92 ± 6.07% baseline); however, there was full recovery of body weight by day 14 (see Supplementary Fig. 3: Figure S3a). RF treatment had no material impact on body weight (see Supplementary Fig. 3: Figure S3b).

### Exercise capacity on a treadmill

**Figure 4:**
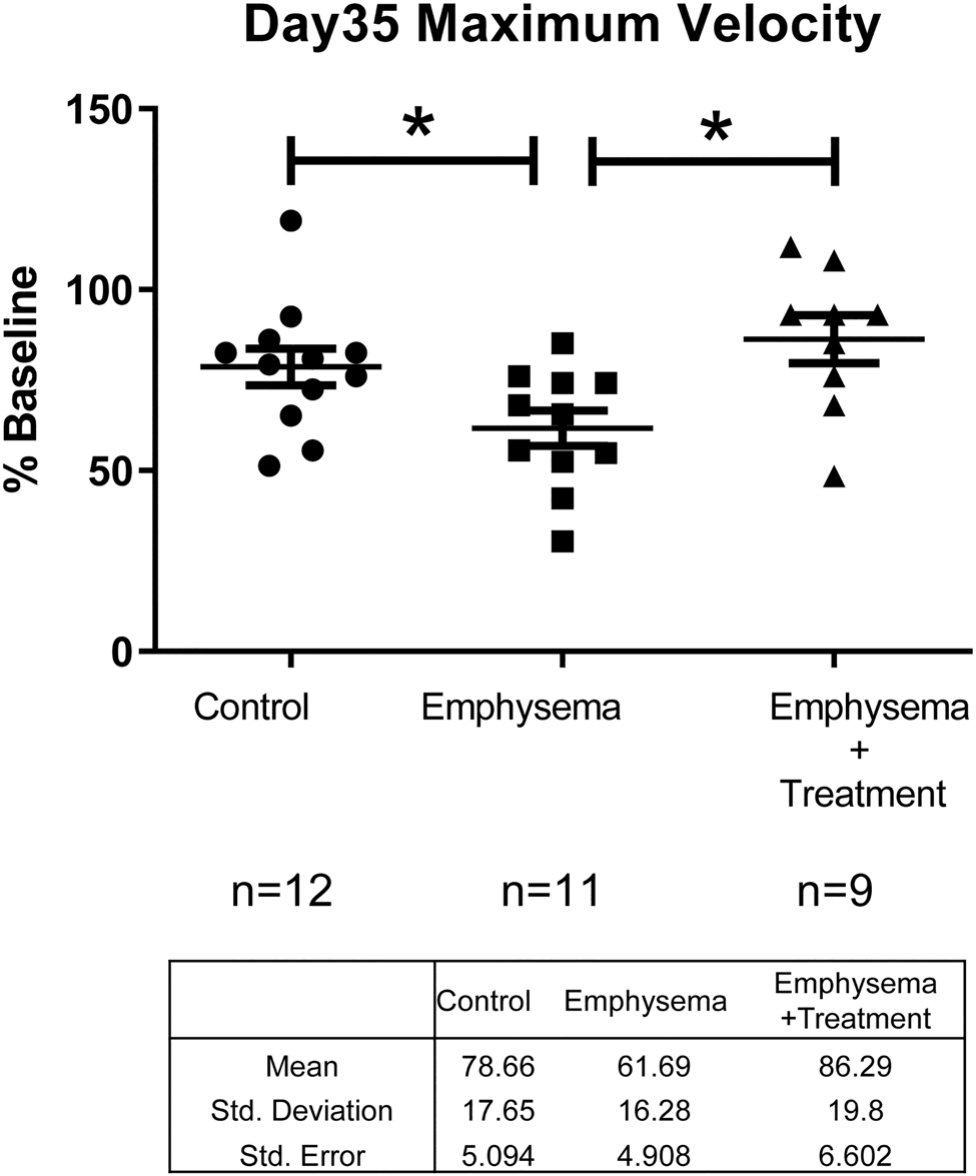
Maximum velocity on day 35. While the emphysema group showed a significant reduction in peak exercise velocity compared to that in the control group, the treatment group showed significantly improved peak exercise velocity compared with emphysema group that represents the therapeutic effect of the RF therapy. Asterisks above the horizontal lines indicate a significant difference in the comparisons between the groups (*p < 0.05).

Although there was no significant difference in the peak treadmill velocity across the groups at day 14 post-instillation (see Supplementary Fig. 4: Figure S4), at 35 days post-instillation (our primary endpoint), mice in the emphysema group demonstrated a significant reduction in peak treadmill velocity compared with those in the control group (61.69% of baseline levels vs 78.66% of baseline levels; p = 0.03) (Fig. 4). RF treatment was able to significantly improve the peak treadmill velocity of mice with emphysema (86.29% of baseline levels vs 61.69% of baseline levels; p = 0.01; Fig. 4), indicating a therapeutic effect of the RF therapy.

### Lung compliance and Lm

**Figure 5:**
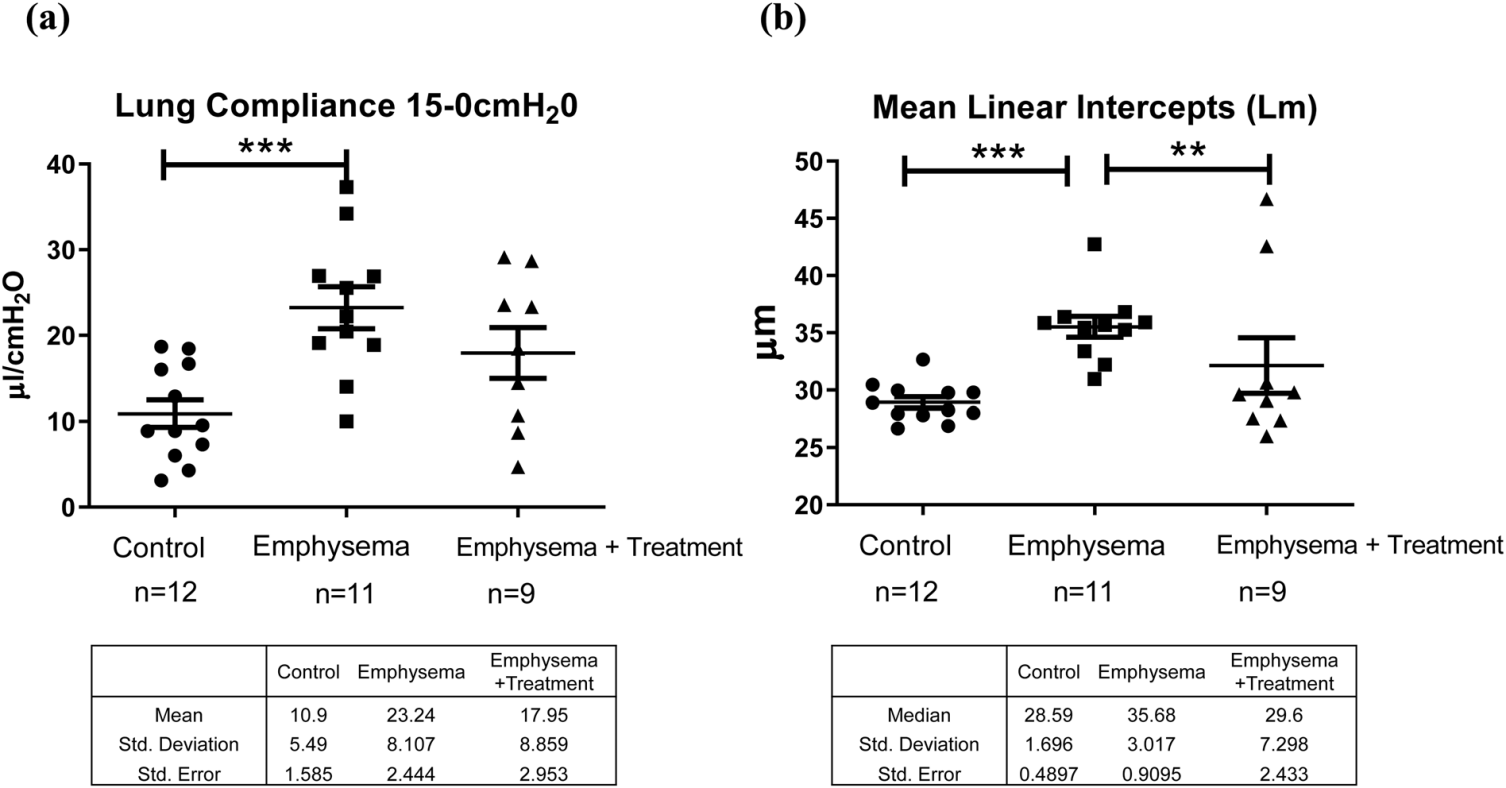
Lung compliance and Lm. (**a**) Lung compliance: The lung compliance was measured by using the water displacement method. Consistent with a decreased stiffness of the lungs, the emphysema group showed a significant increase in compliance compared to the saline group. Lung compliance of the emphysema + treatment group was lower than the emphysema group, albeit not statistically significant. (**b**) Mean linear intercepts (Lm): Compared to the control group, the Lm was significantly increased in the emphysema group while significantly decreased in the emphysema + treatment group. Asterisks above the horizontal lines indicate a significant difference in the comparisons between the groups (**p < 0.01, ***p < 0.001).

The emphysema group showed a significant increase in compliance compared to the saline group (23.24 µl/cmH_2_0 vs 10.90 µl/cmH_2_0, p < 0.01). The lung compliance of the emphysema + treatment group was lower than the emphysema group (18.63 µl/cmH_2_0 vs 23.24 µl/cmH_2_0), although the comparison did not reach statistical significance owing to a limited sample size (Fig. 5a). Lm was significantly increased in the emphysema group compared to the control group, consistent with the presence of emphysema in these animals (median 35.68 µm vs 28.59 µm, p < 0.01). A significant decrease in Lm (median 29.60 µm vs 35.68 µm, p = 0.03) was observed in the emphysema + treatment group compared to the emphysema group (Fig. 5b).

### Fibrosis assessment

**Figure 6:**
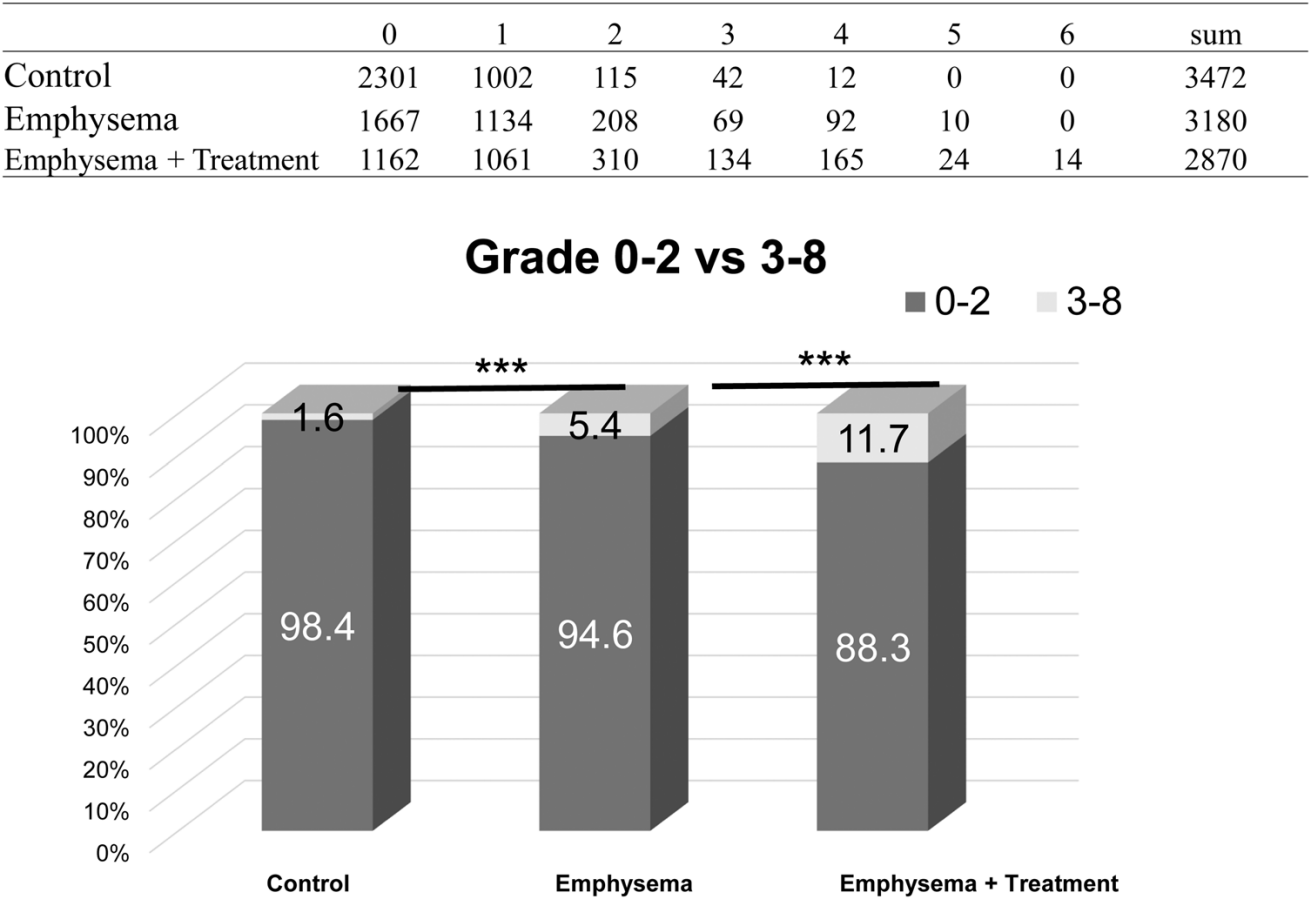
Fibrosis assessment by using the modified Ashcroft scoring. The breakdown of the total 1 mm × 1 mm squares of the specimen are shown in the upper table. The lower graph shows that the emphysema + treatment group had a significantly higher distribution of clear-cut fibrosis (Grade 3 ≧) compared to the emphysema group. Asterisks above the horizontal lines indicate a significant difference in the comparisons between the groups ***p < 0.001).

**Figure 7:**
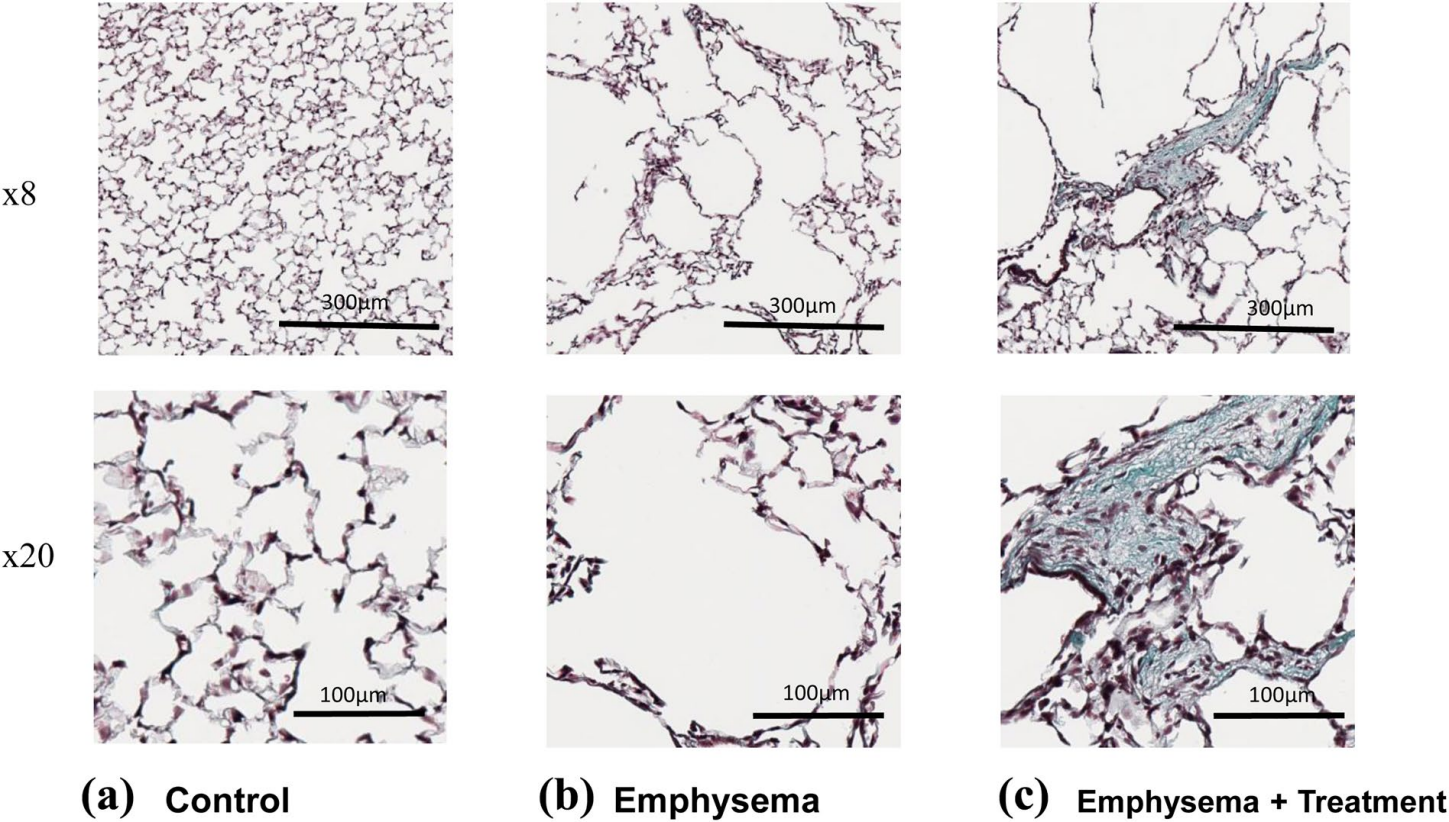
Representative Masson’s trichrome staining photomicrographs showing the morphology of lung. (**a**) Control: saline (100 µl), (**b**) Emphysema: PPE (1.5U/100 g body weight), and (**c**) Emphysema + Treatment: PPE + RF (1.5U/100 g body weight + 0.5 W/g) at day 35. Upper panels ×8 magnification (scale bar 300 µm). Lower panels ×20 magnification (scale bar 100 µm). Clear emphysema was observed in emphysema group and fibrosis was induced by the RF treatment as seen in the emphysema + treatment group.

In total, 9,522 regions were individually assessed per animal by 2 reviewers and the frequency distribution of the grades was plotted for each of the experimental groups. The emphysema + treatment group had a significantly higher distribution of fibrosis (Grade 3 or greater) compared to the emphysema group (5.4% vs 11.7%, p < 0.01) (Fig. 6). As shown in Fig. 7, the emphysema group showed emphysema while the emphysema + treatment group showed mild fibrotic lesions in the emphysematous tissue.

## Discussion

To the best of our knowledge, this is the first study to show that extracorporeal RF treatment improves exercise capacity of animals with emphysema. This suggests that RF therapy may be an effective treatment for patients with COPD. In COPD, static hyperinflation reduces inspiratory capacity and is commonly associated with dynamic hyperinflation during exercise, which leads to increased dyspnea and limitation of exercise capacity. It is thought that hyperinflation develops early in the disease and is the main mechanism for exertional dyspnea^22,23^. In humans, exercise tolerance is a powerful indicator of health status and outcomes of patients with COPD. Limitations in exercise capacity are commonly assessed using either a self-paced walking test^24,25^ or incremental cardiopulmonary exercise testing in a laboratory^26^. To mimic this clinical scenario, we used a treadmill test in mice with emphysema to determine the impact of RF therapy on exercise tolerance; we showed that RF therapy significantly improved exercise capacity of mice with emphysema.

In humans, the most common risk factors for COPD are cigarette smoking and exposure to particulate matter. To mimic this in mice, we instilled PPE intratracheally. The use of PPE produced histological and physiological changes in the murine lungs, consistent with emphysema. After a single elastase treatment, the Lm of these mice increased significantly from 28.6 to 35.7 µm, similar to what has been previously reported in other murine models of emphysema^27–31^. Consistent with these histological changes, ex vivo, these lungs also demonstrated increased compliance. RF therapy led to a significant reduction in both Lm and compliance in the emphysema + treated (versus emphysema alone) mice, similar to what we had previously observed in rats^16^. While the exact mechanism by which this occurs is unknown, we posit that RF therapy induces mild thermal injury in the emphysematous (but not in “normal”) lungs, which in turn leads to inflammation and eventual scarring in the affected regions. Consistent with this hypothesis, we observed in the RF-treated lungs evidence for mild fibrosis.

Notably, RF therapy did not cause any major complications to the vital organs in the treated mice. One notable exception was the skin, which showed evidence of mild thermal injury post-therapy. This complication was mitigated by running cool (distilled) water over the skin of the treated animals during RF therapy.

There were limitations to the present study. First, our emphysema model does not fully replicate pathological or physiological changes that are associated with COPD in patients. As noted previously, in humans, COPD develops slowly over many decades through an inflammatory process that is driven by risk factors such as cigarette smoke or air pollution exposure. Pathologically, it is characterized predominantly by centrilobular rather than panlobular emphysema except in those with alpha-1-antitrypsin deficiency. In contrast, PPE-induced emphysema in mice leads to panlobular destruction of lung tissue as previously reported^32^ and demonstrated in the present study^33^. Nevertheless, a PPE-induced model displays many of the important characteristics of COPD lungs including destruction of alveolar space and “small” airways and increased lung compliance, regional hypoxemia and airflow limitation^33–35^. In addition, this study builds upon the results of our previous study^16^ that used a PPE-induced emphysema model in rats demonstrating the repeatability of this model in rodents. Second, although in this study, we showed a successful improvement in exercise capacity of RF-treated mice with emphysema, the durability of these improvements is not certain. Third, there were significant number of animals that “dropped-out” for a variety of different reasons in strict accordance to the recommendations by the Animal Care Committee of the University of British Columbia, which may have led to selection bias (i.e. survivor effect). This may reduce the generalizability of the results but not their validity. Fourth, we did not collect data on exercise capacity of control mice which were treated with RF because our animal care facility could only accommodate 3 groups for the running experiments on a treadmill. We performed an ancillary study in which mice were exposed to RF treatment and sacrificed 3 weeks later. These mice lungs showed a very low burden of fibrosis. We have included representative images of the lung histology in the supplement (Supplementary Fig. 2). We did not perform a running test because we had limited statistical power in the ancillary study for this endpoint. However, there is no compelling reason to believe that RF therapy would lead to improvements in the exercise capacity of normal mice. Last, although we posit that RF selectively reduces the volume of lung tissue that is emphysematous (because of impaired blood supply), the present study was not designed to address the question of mechanism. Thus, the exact pathways by which RF improves exercise tolerance of emphysematous mice remain largely a mystery. Additional mechanistic studies are needed in the future to answer this crucially important question.

## Conclusion

Here, we showed that extracorporeal RF therapy improves exercise capacity of mice with emphysema. These data highlight the therapeutic potential of RF treatment in improving the functional status of patients with COPD.

## Supplementary Information

Supplementary Information.

## Data Availability

The datasets used and/or analyzed during the current study are available from the corresponding author on reasonable request.

## Abbreviations

COPD: Chronic obstructive pulmonary disease
RF: Radiofrequency
PPE: Porcine pancreatic elastase
US: United States
LVRS: Lung volume reduction surgery
ELVR: Endoscopic lung volume reduction
BLVR: Bronchoscopy lung volume reduction
BTVA: Bronchial thermal vapor ablation
RFA: Radiofrequency ablation
H & E: Hematoxylin and eosin
Lm: Mean linear intercept length
SEM: Standard error of the mean
