# Supplementary material for: Radiofrequency therapy improves exercise capacity of mice with emphysema
Source: Sci Rep. 2021 Oct 8;11:20056. doi: 10.1038/s41598-021-99474-8 (PMC8501094; doi:10.1038/s41598-021-99474-8)
Supplement: Supplementary file 1 — Supplementary Information. [file 41598_2021_99474_MOESM1_ESM.pdf]

Title;

Radiofrequency therapy improves exercise capacity of mice with emphysema

Authors;

Mai Tsutsui[1], Chung Yan Cheung[1], Takeyuki Wada[1], Jen-erh Jaw[1], Cheng Wei Tony Yang [1], Pascal Bernatchez[1,4], Zoe White[1,4], Chen Xi Yang[1], Eun Jeong Annie Bae[1], Lauren H. Choi[1], Dan Gelbart[2], Samuel Lichtenstein[5], Lindsay Machan[2], Eran Elizur[2], Kim Wolff[2], Evan Goodacre[2], Marek Lipnicki[2], Denny Wong[2], Don D. Sin[1,3 *]

1. Centre for Heart Lung Innovation, St. Paul's Hospital, University of British Columbia, Vancouver, BC

2. Ikomed Technologies Inc., Vancouver, BC

3. Division of Respiratory Medicine, University of British Columbia, Vancouver, BC.

4. Department of Anesthesiology, Pharmacology and Therapeutics, University of British Columbia, Vancouver, BC.

5. Division of Cardiac Surgery, University of British Columbia, Vancouver, BC

*Corresponding author;

Don D. Sin

Centre for Heart Lung Innovation, St. Paul's Hospital, University of British Columbia

1081 Burrard Street, Vancouver, BC, V6Z 1Y6 Canada.

TEL: (604)806-8818 FAX: (604)806-9274

E-mail: don.sin@hli.ubc.ca

Supplementary Information

Supplementary Figure 1 (Figure S1). Results of the PPE dose optimization experiment.

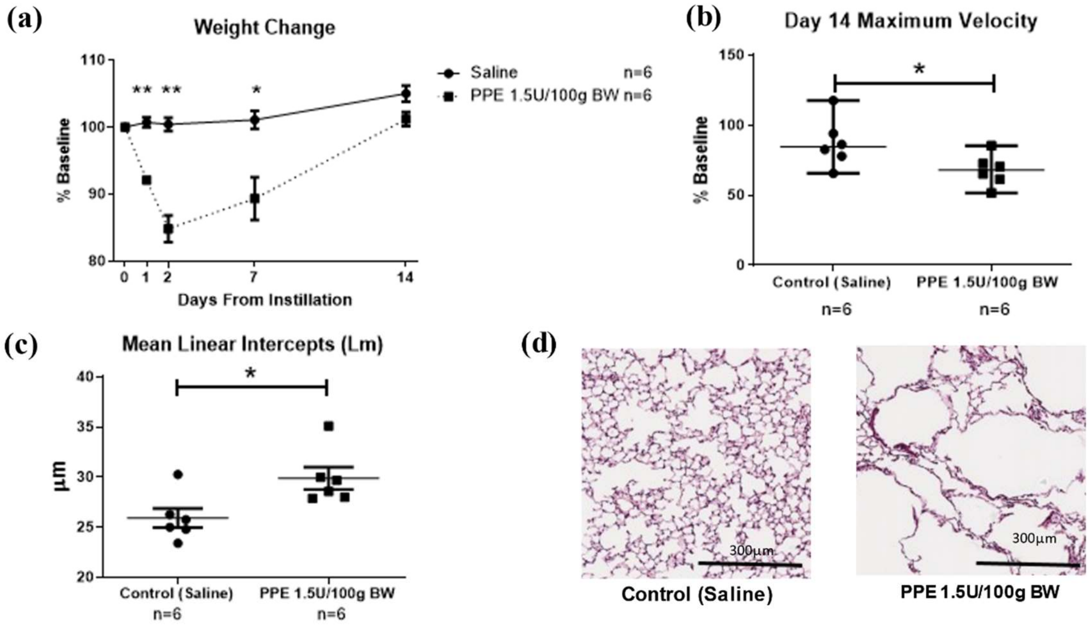

(a) Body weight change after intratracheal instillation. The group instilled with PPE (1.5 U/ 100g body weight (BW)) showed significant body weight loss until day 7 after the instillation. (b) Maximum velocity compared to the baseline on day 14. The PPE instilled group showed a significantly greater velocity loss. (c) Mean linear intercepts were significantly greater in the PPE group. (d) Representative images (H&E staining) of the lung histology on day 14. Clear emphysema was observed in the PPE instilled group.

Asterisks indicate a significant difference in the comparisons between the groups (*$p<0.05$, **$p<0.01$).

Supplementary Figure 2 (Figure S2). Results of the safety investigation of RF treatment

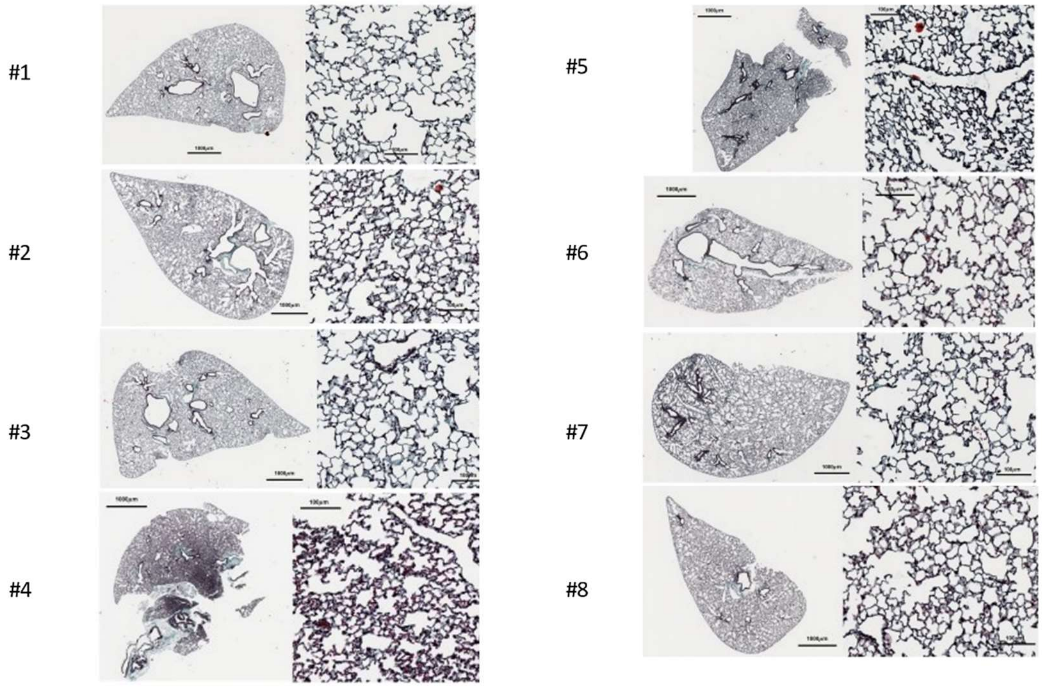

In terms of the control + treatment group, we conducted 3 additional experiments to determine the safety of RF treatment. First, in order to assess the safety threshold of RF power, we performed a dosing experiment in healthy control mice with normal lungs (n=4) using 5W to 20 W (5W, 10W, 15W, 20W) of RF for 1-minute with 3 repeats across 30-second intervals, respectively. We found that 20W was lethal causing acute pulmonary hemorrhage. We also observed overheating of skin when mice were given 15W (though this could be controlled with percutaneous treatment with cold saline). In the 2nd experiment, we applied 15W of RF energy on 5 mice (8 weeks male C57BL/6 mice, Jackson Laboratories). However, the first mice died from acute pulmonary hemorrhage and thus we reduced the RF power to 10W for subsequent

animals and determined that the optimal power threshold to be 0.55W/g body weight. In the third experiment, 8 mice (8-9 weeks male C57BL/6 mice, Jackson Laboratories, identified as #1 to #8) were given 0.55W/g bodyweight of RF (which translated to10.3-14.3W of RF per animal) and observed them for 3 weeks after the treatment. In these mice lungs, fibrosis scores were either none (0) or trivial (1-2) at worst as shown in the figure. Thus, we believe that this dose, which was used in the primary experiment described in the manuscript, is safe and does not induce significant fibrosis.

Supplementary Figure 3 (Figure S3). Body weight of mice over the study period.

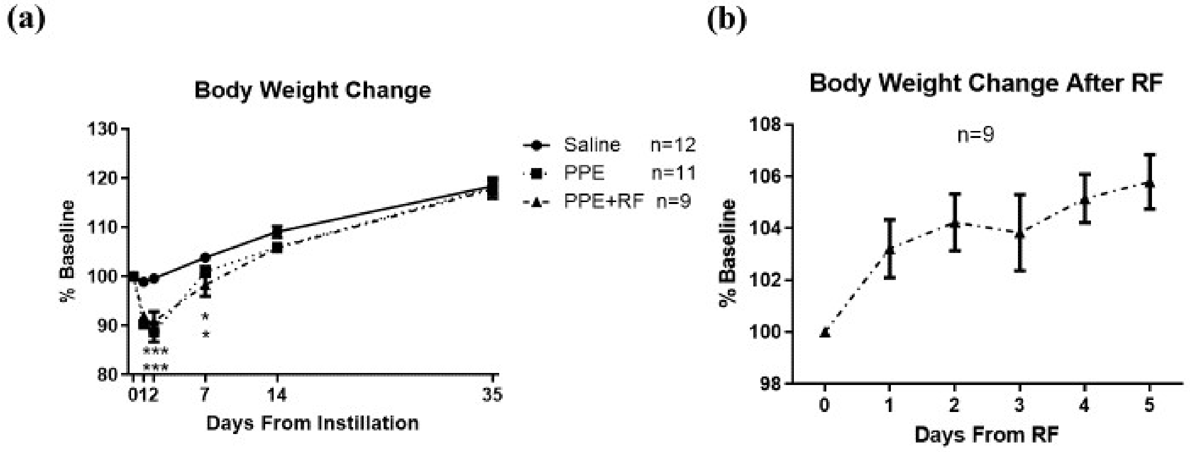

(a): PPE-instilled mice lost body weight compared with saline controls until day 7 post-instillation. The weight loss was at maximum on day 2 and recovered by day 14. The asterisks under each time point indicate significant differences compared to the saline control group (*p<0.05, ***p<0.001). (b): RF treatment did not induce weight loss.

Supplementary Figure 4 (Figure S4). Maximum velocity on day14.

(a)

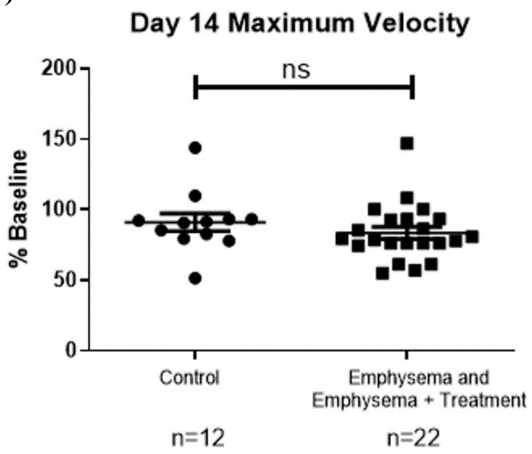

(b)

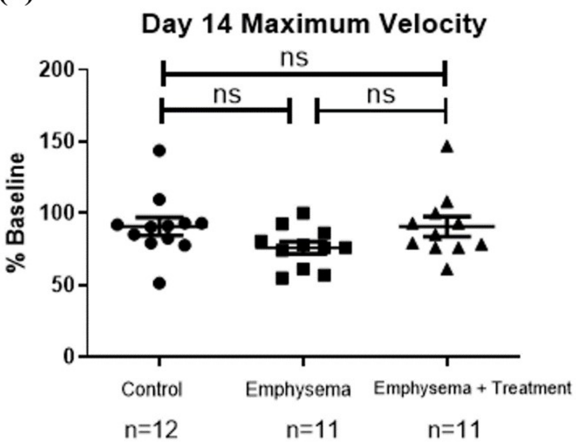

On day 14, the maximum velocity was measured just before the RF treatment. Therefore, the emphysema + treatment group is logically identical to the emphysema group. (a) Maximum velocity on day 14. No significant difference was observed between the control and emphysema and emphysema + treatment group. (b) A detailed comparison of the maximum velocity on day 14. No differences were observed among the 3 groups.

Supplementary Figure 5 (Figure S5). Secondary analysis including the animals which were excluded from the final analysis.

(a)

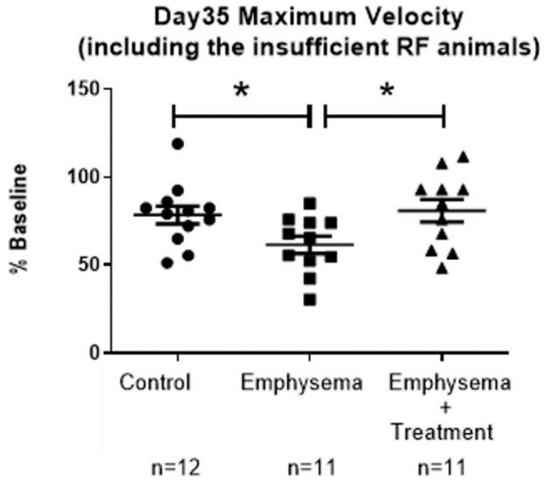

	Control	Emphysema	Emphysema +Treatment
Mean	78.66	61.69	81.09
Std. Deviation	17.65	16.28	21.16
Std. Error	5.094	4.908	6.381

(b)

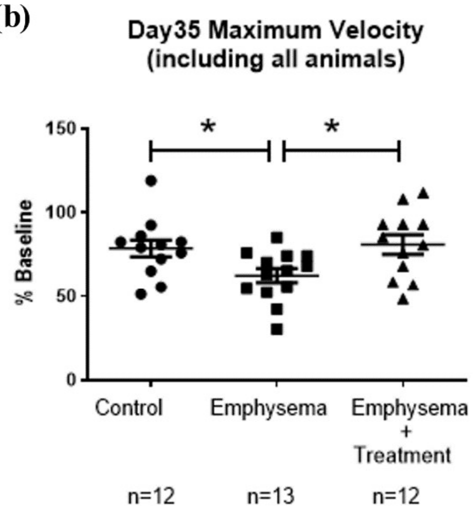

	Control	Emphysema	Emphysema +Treatment
Mean	78.66	62.45	81.06
Std. Deviation	17.65	15.05	20.18
Std. Error	5.094	4.175	5.825

(a) Secondary analysis including the 2 animals that were excluded due to “insufficient RF”. The treadmill data still shows a significant improvement in exercise capacity at day 35 (endpoint). (b) The treadmill data at the primary endpoint including the animals whose data were excluded in the original analysis due to water displacement failure. Inclusion of these animals’ treadmill data does not materially affect the overall conclusions of the study.

Supplementary Figure 6 (Figure S6). Exercise capacity change over time

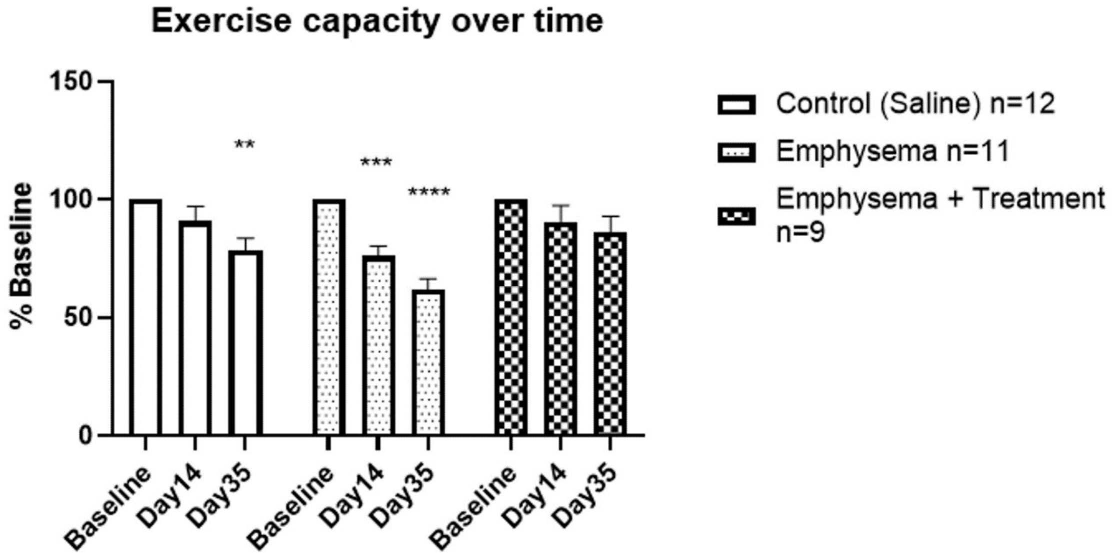

While the control group and emphysema group showed significant exercise capacity decline on day 35, the emphysema + treatment group did not show significant decline. The asterisks shown in the bar graph show the statistically significant difference compared to the baseline in each group (*p<0.01, ***P<0.001, ****p<0.0001).
